# The role of markup for enabling interoperability in health informatics

**DOI:** 10.3389/fphys.2015.00152

**Published:** 2015-05-18

**Authors:** Steve McKeever, David Johnson

**Affiliations:** ^1^Department of Informatics and Media, Uppsala UniversityUppsala, Sweden; ^2^Saint Petersburg National Research University of Information Technologies, Mechanics and Optics (ITMO)Saint Petersburg, Russia; ^3^Data Science Institute, Imperial College LondonLondon, UK; ^4^Department of Computing, Imperial College LondonLondon, UK

**Keywords:** physiological modeling, XML, interoperability, execution environments, patient data

## Abstract

Interoperability is the faculty of making information systems work together. In this paper we will distinguish a number of different forms that interoperability can take and show how they are realized on a variety of physiological and health care use cases. The last 15 years has seen the rise of very cheap digital storage both on and off site. With the advent of the *Internet of Things* people's expectations are for greater interconnectivity and seamless interoperability. The potential impact these technologies have on healthcare are dramatic: from improved diagnoses through immediate access to a patient's electronic health record, to *in silico* modeling of organs and early stage drug trials, to predictive medicine based on top-down modeling of disease progression and treatment. We will begin by looking at the underlying technology, classify the various kinds of interoperability that exist in the field, and discuss how they are realized. We conclude with a discussion on future possibilities that big data and further standardizations will enable.

## 1. Introduction

It has been said that progress is impossible without change and in a digital world it would seem archaic to still have handwritten patient health records. Similarly, the development of new experimental tools, methods, and technologies based on computers has increased our understanding of human anatomy and physiology.

The fundamental theory that underpins computation is that source code and data are interchangeable. However, enabling different systems to exchange such information requires both standards and technologies that deliver viable and meaningful communication. Much of the underlying technology for creating semi-structured data and enabling data exchange was developed in the 1970s. However, it has only been since the late 1990s that their widespread use became feasible due to the dramatic price drop in the cost of storage and the rise of the Internet. A markup language is a standard for annotating a document in a way that is syntactically distinguishable from the content. Early examples of this were created by IBM to annotate documents with formatting commands so that they did not need separate versions for each printing device. In these early cases, the modeling annotations were fixed. The eXtensible Mark Language^1^ (XML) is a set of syntactic rules that allow users to develop their own annotations, and more precisely, their own markup languages.

The ability to easily create, read and modify one's own document structures using a standard template facilitates *syntactic interoperability*. In the case of XML, this is achieved through a hierarchical structure composed of elements and attributes. Alternatively, JSON^2^ adopts a less cluttered format based on attribute key-value pairs to produce a similar result.

Alas the adoption of a generalized markup language is not sufficient to create interoperability. Both sides of a communication need to be able to interpret the information exchanged. To achieve *semantic interoperability*, controlled vocabularies, and standard taxonomies are required. A more generic solution is achieved through the adoption of ontology languages. Such Semantic Web techniques allow the encoding of knowledge about specific domains by augmenting existing documents with attributes that denote meaning. This allows information to be exchanged meaningfully and accurately, even when terms are expressed in different languages, or when two or more terms refer to the same concept but are not easily recognized as synonyms. The Semantic Web Health Care and Life Sciences Interest Group^3^ is tasked to develop and encourage the use of software technologies in the global healthcare arena: from ontologies to integrate biological data, to providing decision support capabilities for patient record systems, and looking at linking the laboratory to the clinic.

Alongside the exchange of information between systems, we require executing computer program components to be able to communicate. If we have two or more components operating over a domain of interest, it would be opportune to pull these resources together in a meaningful manner. This amounts to *execution interoperability*. Computer models have become valuable tools for the understanding of phenomena that govern biophysical behaviors. *In silico* models allow information generated from code that simulates different physical scales to be combined in order to provide a better picture of the coupled processes and structures.

There are different approaches to biological systems modeling (Noble, 2002). A “bottom-up” approach looks at simulating systems from a reductionist point of view, integrating multiple functional components. A “top-down” approach looks at the object in its entirety and develops simulations that match known observations. For example, in modeling cancer both of these approaches are used to simulate different aspects of cancer, such as cancer progression and tumor growth. Naturally there is interest in combining such mathematical modeling techniques in a hybrid fashion. Enabling information exchange between components of compound and hybrid models is not trivial, and this execution interoperability requires syntactic and semantically interoperable approaches.

Take for example computer models of Glioblastoma Multiforme (GBM), an aggressive type of brain tumor. It is possible to combine the two distinctly different modeling approaches to increase the accuracy of a diagnosis. Malignant gliomas are progressive brain tumors. These are classed into anaplastic gliomas and GBM. Patients who suffer from anaplastic gliomas typically survive for 2–3 years. However, the majority of patients with GBM die of the disease within a year after diagnosis (Louis et al., 2007). The recent combined approach of applying temozolomide and radiotherapy has increased the survival period from 12 to 15 months (Minniti et al., 2008). Improvement in life expectancy and quality for patients with GBM is needed. It is now apparent that this can be achieved through collaboration between clinicians, basic researchers, computer scientists, and mathematicians, where many new treatments will be developed with help from personalized computational modeling to increase survival rates and periods.

The demand for greater interoperability from the physiological modeling and health informatics perspective has been largely driven by the European Virtual Physiological Human network^4^ (Hunter et al., 2013). The problem this network is attempting to address is the sharing of the vast but diverse knowledge created by computational biomedical scientists. There are many scientific approaches applied and new emergent technologies but enabling interoperability and reusability is proving to be very difficult. A key source for this disparity originates from the lack of consistent cataloging and annotation of data and models.

This paper will expand on the above through use cases, explaining how markup languages are useful tools to both health informatics and physiological modeling. In Section 2 we will look at how markup is used for electronic health records. In Section 3 we discuss how biological components can be described using markup languages, used to create implementations and form useful repositories. We delve deeper into this theme in Section 4 where we look at what is required to ensure code blocks are extensible and reusable, essential to ensuring interoperability at the model level. We broaden the discussion in Section 5 where we look at how models and data can be brought together through metadata. One of the defining aspects of this field is how heterogenous both the data and models are. Being able to deal with the artifacts in a uniform manner is vital for collaborative efforts. In Section 6 we discuss how top-down and button-up models, from separate repositories, can be linked together to create larger models for the case of modeling tumor progression. Finally in Section 7 we discuss efforts to specify how to perform *in silico* experiments automatically. In Section 8 we summarize and look toward the future.

## 2. Interoperability of patient data

An electronic health record (EHR) is a sequence of health information about an individual patient. It is a digital record that can be shared across different health care settings: over a firewall protected intranet, an enterprize-wide information system, or over the internet. An EHR covers a range of patient data, including personal statistics like age and weight, medical history, medication and allergies, laboratory test results, immunization history, radiology images, vital signs.

In order to ensure semantic interoperability EHRs should adopt a standard taxonomy like SNOMED-CT^5^ (Cornet and de Keizer, 2008) which is a multilingual healthcare thesaurus with an ontological foundation. However, this might not always be possible with legacy systems and data extracted from relational database management systems. One medical center could have neurological evaluation documents with terms such as: “Impairments,” “History of Present Illness,” “Blood Pressure,” “Flexion,” and “Int. rotation.” Another hospital in the same health system may have a totally different clinical documentation system using an alternative convention for expressing the same information. Corresponding tags for similar documents at this hospital may be: “Impairments,” “HPI,” “BP,” “Flex,” and “IR.” If these documents where in XML then those from the hospital could be *normalized*. Normalization is the process of standardizing after the fact. For instance, defining rules like “information following the BP tag from the hospital is the same as the information following Blood Pressure tag from the medical center.”

Assuming semantic interoperability has been achieved, either through the use of standard ontologies or through normalization, health systems can then *aggregate* EHRs for use in downstream functions in ways that were not possible before. Naturally this facilitates data gathering for clinical evidence-based medical practices. It also opens up the door to meta-analysis of clinical trials. Many clinical trials are too small to yield statistically significant conclusions. However, if sufficiently many related trials have been conducted, each investigating a similar medical hypothesis, then the data could be integrated and the results would be more informative. Semantic interoperability in these cases requires more than common ontologies for the physiological markers but also information concerning the context of the data, such as how the data was collected and who collected it, in order for aggregation to be meaningful (Davies et al., 2014). On a day to day basis aggregation also simplifies the administrative workload of health providers.

An outstanding issue is that EHRs are typically stored where the patient is registered, either with a general practice or the local health provider. With the advent of interoperable, secure and trusted means of porting EHRs patient data will not be restricted and we will see an increase in patient care commensurate with greater mobility through traveling and peripatetic work patterns. The challenges posed in implementing EHRs, even with interoperable data formats, should not however be underestimated as demonstrated by the scrapping of the 12 billion (approximately US $18.5 billion) UK National Health Service (NHS) National Programme for IT, the NPfIT (Mathieson, 2011), however structured, coded patient data exists—for example, in EHRs in specific therapeutic areas as well as in more generalized clinical standards—and automatic extraction and normalization of data is increasingly possible. It may not be standard practice universally, but structured EHRs are being adopted. While the NPfIT was scrapped, work is ongoing by the NHS to adopt localized EHR solutions in individual local health providers (Sheikh et al., 2010).

## 3. Separating the model from the code

Scientific modeling aims to capture features of the world that we wish to understand, quantify, visualize or simulate. Since the 1960s computers have been used to model biological processes with the aim of understanding and predicting diseases. Biological systems involve many processes occurring in parallel over a wide range of time scales and size. Organs such as the heart are comparatively large and operate over seconds, whereas smaller processes within cells can operate at the nanosecond time scale and are important for modeling tumors. Computational biology is becoming ever more accessible due to the dramatic increase in computing power over the last two decades. The result of this is we can now apply computer simulations to track increasingly detailed descriptions of cells and model large numbers of cells at the same time. Moreover, we can now couple a wide range of scales into the same simulation to allow more complex biological structures to be modeled. The multi-scale and multi-physics nature of these models makes their instigation non-trivial, both from the mathematical and biological perspectives. Sharing and reusing models has proved tricky. The published models are hard to verify and often lack information that is required to reproduce the results. It is not uncommon for there to be errors in the papers, both in the mathematical equations but also in the large parameter lists required to generate the models.

The International Union of Physiological Sciences Physiome Project^6^ was created in 1997 with the goal of addressing these issues by providing a framework for the modeling of the human body. Hunter (2007) As part of this project, the specification of the CellML^7^ markup language were released in 2001. It is a language used to store and exchange computer-based mathematical models. Developed out of the cardiac modeling community, CellML aims to cover a range of biological phenomenon, chiefly cell-function. The notation and it's tool base is described in Garny et al. (2008). Simultaneously a separate consortium developed SBML^8^ over a series of workshops and was released in 2002. SBML is also an XML based markup modeling language and was developed to capture bio-chemical processes at the molecular scale.

Other notations have proceeded, each with constructs suitable for the physiological field that they're aiming to emulate but the essential idea remains the same: developing a notation that is domain specific and separate from a general purpose programming language or a mathematical solving tool such as MATLAB; provide means of editing, storing, and simulating these models. For instance, the Pharmacometrics Markup Language^9^ (PharmML) has been designed as the exchange medium for pharmacometric models driven in part by the success of SBML; FieldML^10^ (Christie et al., 2009; Britten et al., 2013) proposes a standard for modeling the physics of structures and fields in physiology such as muscle fibers in heart muscle, as well as linking to other scales of model; and NeuroML^11^ (Goddard et al., 2001; Gleeson et al., 2010) for modeling biophysical and anatomical properties of the neuron and brain.

Having effective physiological markup notations has led to MIRIAM (Minimal Information Required In the Annotation of Models), a community-level effort to standardize the curation and annotation processes of biological models (Novère et al., 2005). MIRIAM consists of a set of guidelines that can be applied to any structured format. Thereby facilitating diverse groups to collaborate and share resulting models. Compliance to these guidelines enables the sharing of software and service architectures built upon modeling activities.

Alongside community guidelines one also needs to include in the model description information that puts the model into a wider context. This information is called metadata, namely “data about data.” These physiological markup notations require metadata for two main reasons:

- To enable **reuse**. If a modeler wants to use a model written by someone else then they need to know about the phenomenon the component describes, such as what biological entity it represents. Where possible these metadata annotations should link with publicly accessible ontologies of such concepts such as the Gene Ontology^12^ (Ashburner et al., 2000), the PROtein ontology^13^ (Natale et al., 2007) or UNIPROT^14^ (Apweiler, 2010). The modelers might also need to know when the model was created and from which experimental data sets it was validated with.
- To enable **curation**. Metadata provides a means for locating particular models and components. It is also important to document the model and binding this information with the model itself keeps the metadata from becoming obsolete as the model is refined.

Sitting on top of these efforts is the BioModels Database^15^ (Li et al., 2010). This database is more than just a repository of models. It contains many manually curated models enriched with semantic meta-data and cross-referenced from external data repositories such as publications. The models, their controlled annotation and all related information is stored in a set of MySQL tables. The database allows scientists to search, store, and retrieve mathematical models. It supports the automatic processing of CellML and SBML files and has an inbuilt SBML simulator.

Monolithic simulation codes written in efficient but poorly engineered programming languages led to a “model engineering crises” centered around initial designs which lacked extensibility, reproducibility, and modifiability. The use of markup has brought transparency, curation and powerful model databases that allow for some degree of interoperability. Model composition will always lag behind biological model discoveries for a number of technical and practical reasons. Modelers have tended to consume available computational resources so being able to run multiple models concurrently, with extra demand on processing speed and memory, will require further resources which might not be readily available. Moreover models are typically developed and validated in isolation. Composing such sub-models to create larger scale models remains problematic. The number of parameters and their range of scales are rarely compatible, and whilst the sub-models might have been validated with respect to some known data, their combination will also need to go through this process. Appropriate data for the composed model might not exist or be sufficiently well-understood to enable rigorous validation to proceed quickly.

## 4. Interoperability from a software engineering perspective

Despite the successes of the markup language efforts described in the previous section, issues arise when attempting to expand on the original notations. Extensibility is a characteristic of systems design. It is a measure of the degree and effort to which a system can introduce new functionality, with minimal disruption to its existing behavior. Both SBML and CellML encapsulate internal components and models to a certain degree. They use relatively simple techniques to ensure backwards compatibility with older models. Complex models which simulate multiple processes achieve this by either spreading the biological concepts over different parts of the code, or by representing multiple concepts in one portion of the code. Neither CellML or SBML provide a means of allowing direct connectivity of data between modules. Thus, any notion of cohesion is not directly supported. The grouping of concerns to achieve better modularity and encapsulation is left to the developer.

Standard module features have been added to CellML and these enable the embedding of sub models. Current work is looking at dealing with variation and stochasticity. If you are solving an ordinary differential equation based model then these features will allow the representation of any model type you like within a CellML framework.

Paralleling traditional software engineering in which one accepts that the system will be extended beyond current considerations, designing systems based on high cohesion and low coupling will ensure future rewrites are mitigated. Coupling is the degree to which each program component relies on each one of the other components, whereas cohesion refers to the degree to which the elements of a component belong together. By ensuring a computer system is designed with low coupled components, with each component displaying high cohesion, then the software engineering goals of high readability, extensibility, maintainability, reusability are better supported. Another way of looking at it is that entities and methods that are distinct should be kept apart while those that are similar should be close together. Low coupling is typically achieved by designing components to interact through well-defined interfaces independently from their internal representation, making them easier to reuse and extend. High cohesion occurs when software is designed to encapsulate functionality that is closely related, making components code easier to understand and maintain.

Using abstraction techniques from modern programming languages such as Generics and well-engineered Inheritance, we show in McKeever et al. (2013) how they lead to reusable and interoperable components through low coupling and high cohesion. We demonstrate their utility on two case studies. Generics were used to parameterize heart models on their ion channels, allowing a range of previously distinct models to be aligned. We used class inheritance to enable run-time substitutability of various tumor growth model components. This enables modelers to easily customize and extend existing models in an intuitive way. Finally we showed that, when combined, these techniques allow model designers to pick and choose suitable abstractions to ensure that their codes may be maintained and extended in a well-structured and type-checked manner.

Well-designed object orientation enables interoperability with a weak semantic alignment at the code level; through interfaces, subtype inheritance, and generic instantiation. However, there is a cost involved with utilizing these techniques; modelers have to spend time and effort designing their code with such abstract architectures in mind in order to reap the benefits. Future work should consider the use of ontologies to facilitate a Model Driven Approach using UMLs meta-object facility and corresponding tool support.

## 5. Semantic interoperability for biomedical data and models

Ensuring that the metadata can actively contribute toward interoperability is the driving force behind the European RICORDO^16^ project (de Bono et al., 2011). Here the focus is on supporting the VPH community through the development of a multiscale ontological framework to enable interoperability amongst its modeling and data resources. The motivation is based on the belief that industrial and clinical traction cannot be achieved unless sharing and reasoning over metadata can be demonstrated in practice. RICORDO draws together a number of key databases at the UK-based European Bioinformatics Institute along with tools and methods. The focus was to create a metadata framework that enables multi scale biological entities to be coupled.

RICORDO exploits standard reference ontologies, that encapsulate biological meaning, in the models metadata to promote interoperability. RICORDO has developed an architecture based on the storing and inference-based querying of their annotations (Wimalaratna et al., 2012). This infrastructure consists of a repository, an intelligent database and a set of applications.

This work has been further refined in the ApiNATOMY^17^ system, where the authors have developed a tool that automatically generates consistent anatomy diagrams and superimposes anatomy-related information (de Bono et al., 2012). A key goal of their on going effort is to support the open biomedical community to collaborate, share and interact with complex data and models in genomics, physiology, pharmacology, and pathology.

Other examples of open semantic standards and ontologies include MAGE-TAB^18^ (Rayner et al., 2006) (MicroArray and Gene Expression Tabular), ISA-TAB^19^ (Sansone et al., 2012), BioPAX^20^ (Biological Pathway Exchange) (Demir et al., 2010), and the Gene Ontology (GO) (Ashburner et al., 2000) to name but a few. Ontologies facilitate machine processing, standardization of resource metadata, as well as reasoning. They enable the navigation and querying of annotated repositories using formalized biomedical knowledge. The ontologies allow for a uniform means of accessing models and data over a wide range of disparate domains.

## 6. Execution platforms

Models and their implementations need to also interoperate with different computational execution platforms (to be able to run a simulation) as well as enabling models to interoperate with each other in combination. As mentioned previously, the simulation of GBM *in silico* is one novel treatment modality by modeling tumor growth and reaction to treatment (Johnson et al., 2013c). Cancer is a phenomenon that occurs at many scales and in order to reliably predict cancer progression over time, including predicting responses to simulated treatment, several scales should be simulated concurrently, and in combination. Different research groups focus on different scales and contexts of tumor dynamics. Fusing modularized models is not trivial, where the fusing of a bottom-up approach with a top-down approach may combine data from subcellular systems biology, DNA methylation status, deregulated metabolic pathways, or size of tumor based on imaging.

To address the need for being able to fuse different tumor models, we developed an XML-based markup language targeted at the tumor modeling domain, TumorML (Johnson et al., 2010, 2011, 2012, 2013, 2014; Sakkalis et al., 2012, 2014). The TumorML XML schema^21^ was developed out of the European Commission Transatlantic Tumor Model Repositories project (TUMOR). The schema allows us to make records of the metadata relating to cancer model descriptions as TumorML XML documents. TumorML inherits elements from a number of other XML standards. Dublin Core is used for basic resource curation that enable some search and provenance (elements such as title, creator, description, publisher, contributor, and date) (Dublin Core Metadata Initiative, 2012). BibT_E_XML is a representation of the BibT_E_X format for bibliographic referencing (Gundersen and Hendrikse, 2007). Reference elements contain a title, source containing a URL, creator, full text citation, and a type that categorizes the reference using BibT_E_XML categories. Abstract model descriptions are used to describe the executable run-time interfaces to cancer models (and cancer model components) as “black boxes,” where XML declarations of input and output parameters describe how data flows in and out of a model. The Job Description Markup Language (JSDL) (Anjomshoaa et al., 2005) is used to describe the basic system (both hardware and software) required to execute TumorML-packaged implementations.

As described in Johnson et al. (2011), models can be either “simple” or “complex,” and this is reflected in the XML schema by having a choice of two patterns that can be enclosed within a model XML element. To recap, a simple model description allows a single computational cancer model to be parceled up, while a complex model describes a compound model; a combined entity made up of simple or complex models enclosed in TumorML.

**Figure 1:**
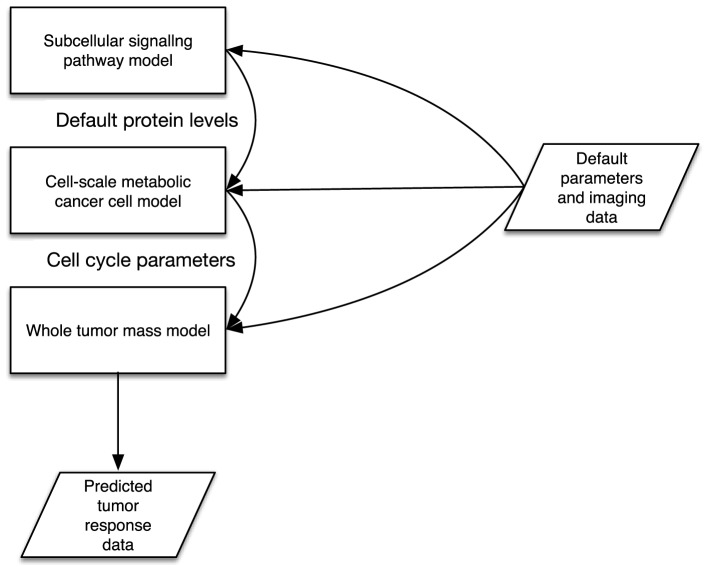
**Illustration of multiple cancer models connected together via some interoperable interfaces**.

A simple model consists of two key descriptors: an input and output parameter specification, described with a parameters XML block with in and out elements that define input and output parameters. Parameters may refer to system-level files in order for implemented models to read in or write out data. Following this, at least one implementation block is used to describe the metadata of a model's software implementation. An implementation specification describes the files that make up the model implementation (e.g., Binary executable program files, source code, initial data/parameter files etc.), as well as instructions on how to handle packaged files, and the minimum software and hardware requirements for running the model. The key enabler of interoperability is in defining the parameter interfaces in a standard way using XML. A complex model is similar to a simple model, however contains multiple model declarations. We then describe a set of instances and a topology of linked sub-model parameters that are connected. This is illustrated in Figure 1, where the example illustrates three models that make up a complex model. The input and output parameters are described in XML in a standardized form as defined in the TumorML schema. in this diagram we show a complex model that is composed of three different models simulating different scales and aspects of cancer biology. Connected input and output parameters must match in terms of computational and semantic compatibility to enable parameter/data passing between component models. This allows for interoperation between linked component models since their input and outputs are declared in the same way.

The Multiscale Modeling Language, MML; and its XML version, xMML; proposes a standard for specifying both multi-scale models and how to couple models of differing simulated scales. The XML markup for building complex models in TumorML is inspired by xMML. This allows computational engines to interpret and execute cancer models, including communicating input/outputs to the process as well as facilitating inter-model communication. Full details on the XML markup used in TumorML can be found in Johnson et al. (2013)

## 7. Experimental platforms

In Section 3 we looked at how to standardize the description of models to ensure reproducibility of simulations. We saw how the MIRIAM (Novère et al., 2005) guidelines enabled the sharing and reuse of models. In this section we will discuss how a second set of minimal information guidelines called MIASE (Waltemath et al., 2011a) (Minimum Information About a Simulation Experiment) specifies the requested information about simulation setups. The key idea is that model reuse can be improved if models and associated data are considered together. The reason for this is that to represent increasingly complex biological phenomena requires models to be instantiated using different conditions, and these conditions must be formally described together with the model itself. A coherent and reproducible means of representing *in silico* experiments is necessary in order to verify or refute a hypothesis.

The MIASE guidelines are a community effort to identify the minimal information necessary to enable simulation experiments to be reproduced. Consequently, the MIASE Guidelines list the information that a modeler must provide so that a numerical simulation experiment, based on a set of quantitative models, can be executed in a manner that others may be able to arrive at the same results.

The guidelines are respected by the Simulation Experiment Description Markup Language (SED-ML ^22^), an XML-based format for simulation experiment encoding (Waltemath et al., 2011b). A SED-ML document would list the models required for an experiment, the transformations needed to be applied to the models before they can be used, the simulation procedures required to run on each model, functions to analyse the results and which ones should be outputted, and finally how the output should be presented. Each of these descriptions do not depend on the underlying implementation of the model. By being model agnostic means that one can perform the same experiment on differing implementations that aim to model the same phenomena.

As SED-ML is a software-independent format describing how to perform simulation experiments, it is not bound to any simulation environment or tool. In Waltemath et al. (2011b) the authors demonstrate that, as the support for SED-ML has grown within the biological modeling community, it has become feasible to exchange simulation descriptions so that the same experiment can be run on different simulation tools. One important use case for SED-ML is functional curation, based on the idea that when mathematical and computational models are being developed and curated the primary goal should be the continuous validation of those models against experimental data. An extended version of SED-ML enables this tight coupling so that the two data sets (experimental and simulated) can be curated together, and as new competing models of the same biological system are developed they can then be compared directly with existing models through use of the same protocols. So from a behavioral point of view, functional curation enables models to be extended and re-used by other members of the community with confidence. This technique has been demonstrated on cardiac electrophysiology cell models (Cooper et al., 2011).

There are many tools that have been developed to perform simulations based on markup descriptions of data and models, for example: CellDesigner, Systems Biology Simulation Core Library, Repose, Flint and CMISS, to name but a few. Beyond a single-cell simulations for example, CellML has been integrated into OpenCMISS (http://www.ncbi.nlm.nih.gov/pmc/articles/PMC4283644/), and other markup languages have been developed for different aspects of physiological simulation (FieldML/PHML/NeuroML). Also within the euHeart project (Smith et al., 2011) some of these standards were even used in generating whole-heart simulation. Multi-scale componentized simulations/computations have been done, perhaps are not yet commonplace, but is a rich and growing area of research in computational biology.

## 8. Conclusion

We have discussed how markup languages have enabled better use of digital technology in the health care and modeling domain through increased interoperability. However, it is still early days in this endeavor. Greater standardization and trusted secure communication will revolutionize patient treatments and ensure greater healthcare provision through healthcare providers having efficient access to patient data anywhere and at any time; automatically generated tool support for clinical trials (Davies et al., 2014); availability of online psychological treatment and support^23^; computational modeling of diseases to personalize medication^24^ and treatment. While EHRs on their own may not provide granular enough detail for biophysical simulations, such simulations would not be considered using only EHR data. Simulations are increasingly being developed that include extremely rich dataset combinations that use EHRs with various kinds of 'omics (genomics, transcriptomics, metabolomics etc.), generalized observed parameters from published literature, and increasingly sensor data such as environment and location, actimetry, and even physiological signal profiles (e.g., EEG/ECG). All of these sources of data require syntactic and semantic means toward interoperating across clinical and research systems.

From the perspective of empowering patients, one can envisage a future in which doctors prescribe mobile *apps* that work with the patient to gather data from smart devices, model disease progression in real time and calculate medication accordingly; with remote access to medical backup to ensure patients are receiving the optimal treatment possible.

We have seen how repositories, such as the BioModels database, provide curated and reusable components that capture a wide range of biological systems. They enable *in silico* experiments to be undertaken without having to implement the models from scratch, solely using the equations and data extracted from the literature. From the modeling perspective much work remains, integrating models over time and space requires considerable effort and new techniques need to be developed in order to link the cell level to the atomistic one for instance. However, genomic sub-models are being introduced into cell models (Niederer et al., 2012) where relevant. Crucial to the successes of model markup languages has been the role of active communities of tool developers and modelers who have fostered the early stages of these projects, many as part of their doctoral studies, to ensure the methodologies developed sustainable momentums within their respective fields.

**Figure 2:**
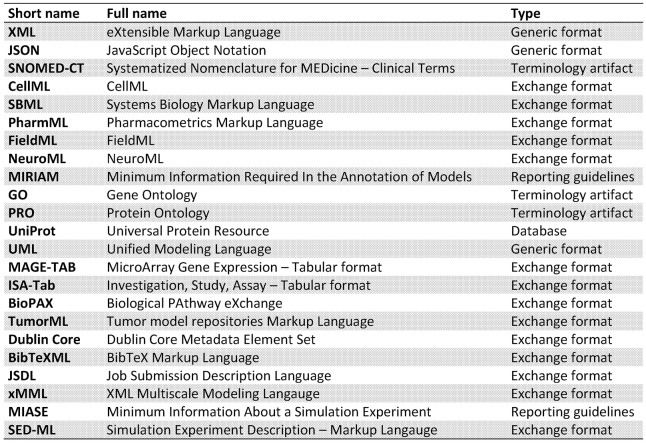
**Summary of different standards covered**.

**Figure 3:**
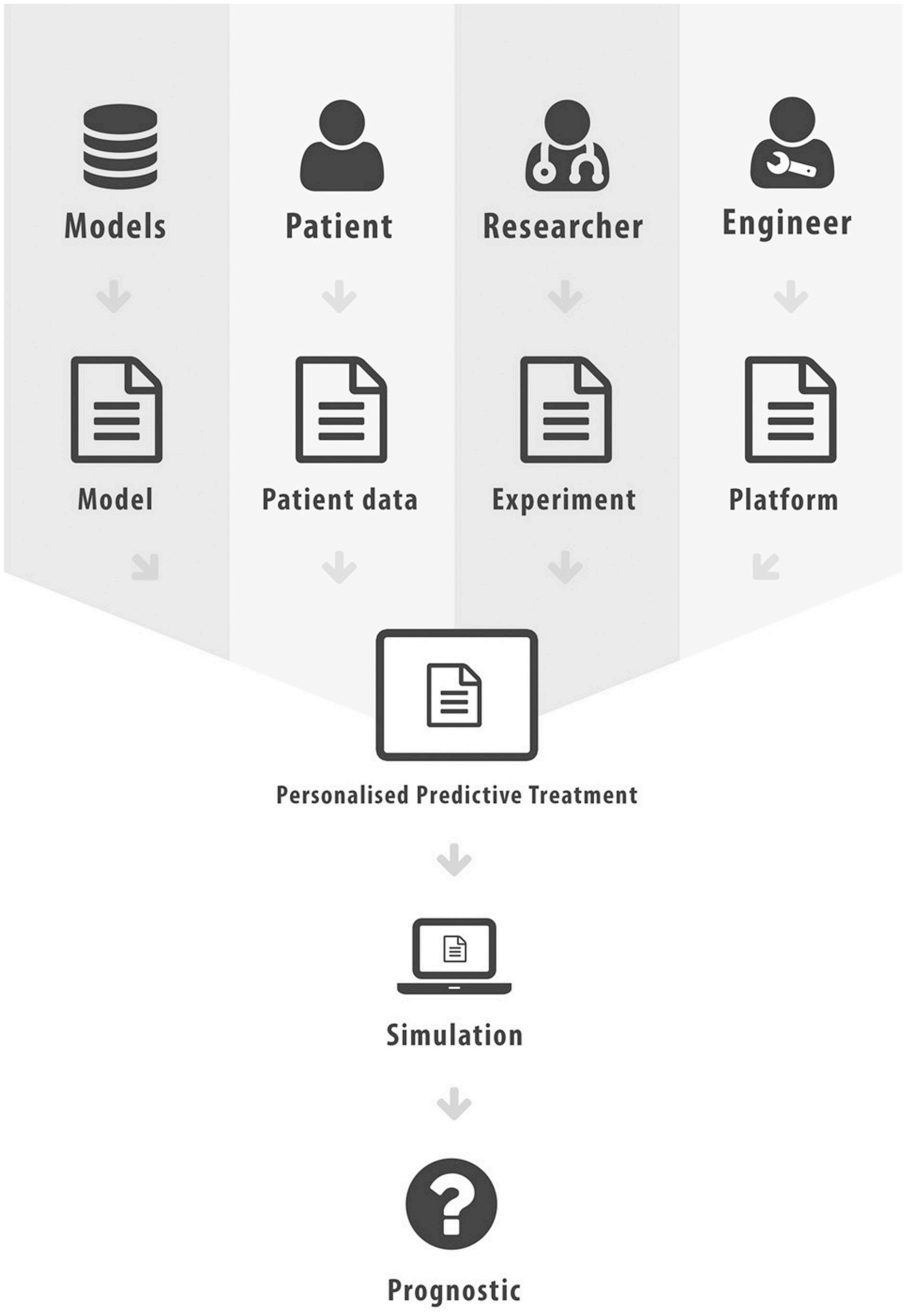
**Facets of Interoperability, where mark-up has been used to characterize each distinct component**.

The aim of this paper was to elucidate some of the different forms interoperability can take in the realm of scientific computing and health informatics. This has not been an exhaustive presentation, more an overview of some key research areas and the potential added value that can be achieved through interoperability. In Figure 2 we list the various different standards that we have discussed in this paper. Figure 3 attempts to portray the various facets of interoperability discussed within this paper. The *patient data*, discussed in Section 2, is the foundation on which health informatics is built. Predictive therapies will require personalized simulations. Such simulations are constructed from *models* described in Sections 3, 4, 5. Many of these models can currently run through community based environments. However, compound models require sophisticated *execution platform* descriptions to enable disparate models to be combined as discussed in Section 6. Finally there are generic frameworks that enable *experiments* to be specified so that models can be run in a reproducible manner as shown in Section 7. We hope to have shown how mark-up languages play an important part in structuring data, describing models, specifying workflows, and creating libraries of experiments in order to combine resources and encourage re-use. A more comprehensive list of biological sharing initiatives can be found at BioSharing^25^.

### Conflict of interest statement

The authors declare that the research was conducted in the absence of any commercial or financial relationships that could be construed as a potential conflict of interest.

## Footnotes

^1^http://www.w3.org/XML/

^2^http://json.org

^3^http://www.w3.org/2011/09/HCLSIGCharter.html

^4^http://vph-portal.eu

^5^http://www.ihtsdo.org/snomed-ct/

^6^http://physiomeproject.org

^7^http://www.cellml.org

^8^http://sbml.org

^9^http://www.ddmore.eu

^10^http://physiomeproject.org/software/fieldml

^11^http://www.neuroml.org/

^12^http://www.geneontology.org/

^13^http://pir.georgetown.edu/pro/

^14^http://www.uniprot.org/

^15^http://www.ebi.ac.uk/biomodels-main/

^16^http://www.ricordo.eu

^17^http://apinatomy.org/

^18^http://fged.org/projects/mage-tab/

^19^http://www.isacommons.org

^20^http://www.biopax.org

^21^http://github.com//tumorml/TumorML-schema

^22^http://sed-ml.org

^23^http://www.u-care.uu.se

^24^http://www.p-medicine.eu

^25^www.biosharing.org/standards
